# Supplementary material for: LSD reconfigures cortical dynamics through faster brain rhythms and increased fractal dimension
Source: Commun Biol. 2026 Oct 7;9:1244. doi: 10.1038/s42003-026-10629-7 (PMC13646281; doi:10.1038/s42003-026-10629-7)
Supplement: Supplementary file 1 [file 42003_2026_10629_MOESM1_ESM.pdf]

LSD Reconfigures Cortical Dynamics Through Faster Brain Rhythms and Increased Fractal Dimension

Venkatesh Subramani[1,2,a], Timothy Nest[1], Annalisa Pascarella[3], Jérémy Brunel[1,4], Yorguin José Mantilla Ramos[1,8], Yann Harel[1,5], Suresh Muthukumaraswamy[6], Robin Carhart-Harris[7], Giulia Lioi[2], Nicolas Farrugia[2], and Karim Jerbi[1, 8, 9, b]

[1]Cognitive and Computational Neuroscience Laboratory (CoCo Lab), Psychology Department, Université de Montréal, Québec, Canada
[2]IMT Atlantique, Lab-STICC, UMR CNRS 6285, F-29238, Brest, France
[3]Institute of Applied Mathematics "M.Picone" - CNR, Italy
[4]Centre de recherche Hôpital Maisonneuve-Rosemont (HMR), Montreal, QC, Canada
[5]CRIUGM (Centre de Recherche de l'Institut Universitaire de Gériatrie de Montréal), Montréal, QC, Canada
[6]University of Auckland, Auckland, New Zealand
[7]University of California, San Francisco, San Francisco, United States
[8]Mila (Quebec AI Institute), Montréal, Québec, Canada
[9]UNIQUE Center (Quebec Neuro-AI Research Center), Montréal, Québec, Canada
[a]svenkatesh.95@gmail.com
[b]karim.jerbi@umontreal.ca

June 1, 2026

---

1 Supplementary Material

1.1 Subjective Reports data

We present here the raw phenomenological scores across the drug and task conditions (Supplementary Fig. S1). The scores were statistically compared using paired t-tests, followed by FDR-correction to account for all the pairwise comparisons.

1.2 Similar topography in spectral features when fitting Specparam up to 120Hz and upto 45Hz

To assess the robustness of our spectral parameterization models, we additionally fit the models using a narrower frequency range (1–45 Hz; Supp. Fig. S2). The first row summarizes

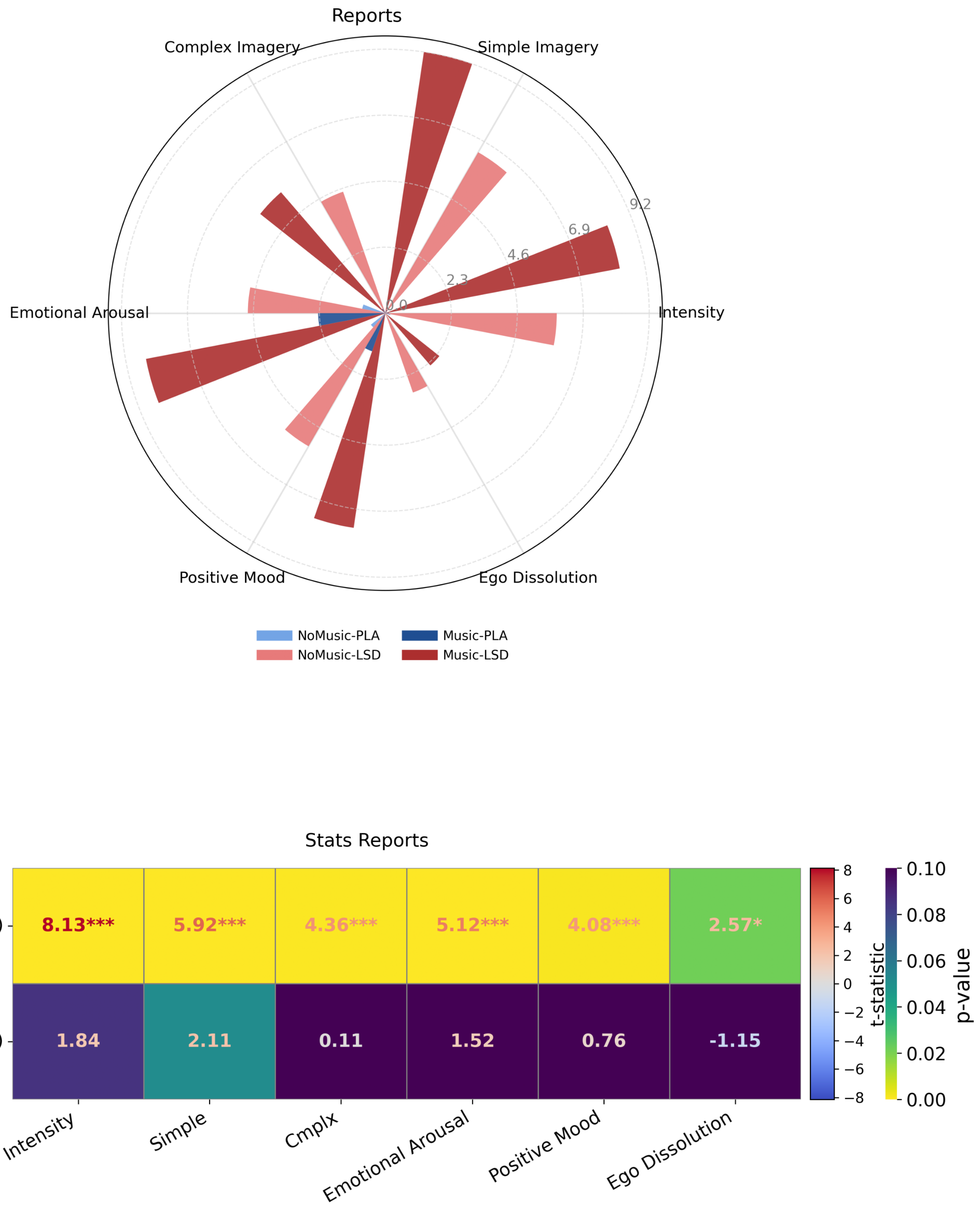

Figure S1: In-scanner subjective ratings across conditions. First and second row respectively correspond to grand-averaged scores and the paired statistical comparisons between LSD and PLA conditions ($p < 0.05$, FDR-corrected).

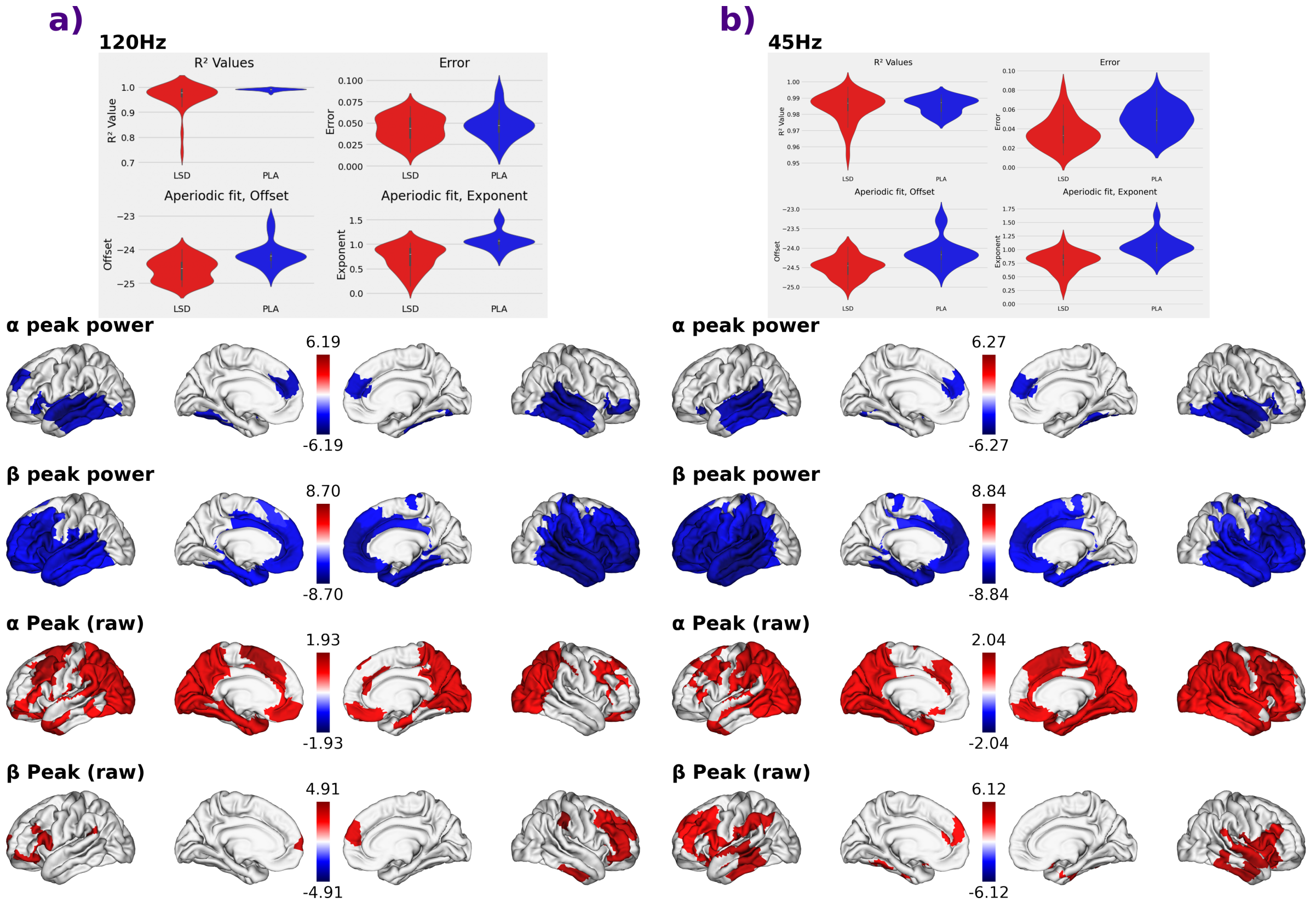

Figure S2: Performance of spectral parameterization models across frequency ranges. **a-b** Global (region-averaged) FOOOF model performance for the broadband (1–120 Hz; a) and narrowband (1–45 Hz; b) spectral ranges. Violin plots show model fit quality (R^2 and error) and aperiodic parameters (offset and exponent) under LSD and PLA. Error rate, R^2, Offset and Exponent of the global (i.e. region-averaged PSD) specparam model (top row) for the frequency range of 1-120Hz (**a**, same as in the manuscript), and for the narrower frequency of 1-45Hz (**b**). Spatial maps under the barplot show the corresponding LSD-PLA contrast maps for α and β Peak Power changes, alpha and β peak shifts across two frequency ranges. Maps show the magnitude and direction of LSD–PLA contrasts, illustrating that the qualitative spatial patterns of peak-power suppression and peak-frequency shifts are replicated across both parametrization ranges.

model performance on the global spectra, with violin plots showing the distribution of R^2, fitting error, offset, and exponent across subjects and conditions. The lower panels display the significant contrast maps for Peak Power and Peak Shifts derived from the narrowband fits. Notably, these maps exhibit highly similar spatial patterns to those obtained with the full 1–120 Hz model, indicating that our main findings are stable with respect to the chosen frequency range and not driven by high-frequency components.

1.3 Oscillatory peaks present most reliably in alpha and beta bands

To determine whether oscillatory peaks observed across subjects reflected meaningful structure rather than chance detections, we applied a binomial test against a theoretical false-positive rate of 0.05. For each parcel and frequency band, oscillatory peaks identified by the spectral parametrization model were binarized for each subject (peak present or

absent). Because each subject has an estimated 5% probability of exhibiting a spurious peak, the number of detected peaks across our 17 subjects can be modeled as a series of independent Bernoulli trials with a null probability of 0.05.

For each parcel, we counted how many subjects showed a peak and then asked: What is the probability of observing at least this many detections purely by chance? This probability is given by the upper tail of the binomial distribution (also known as the survival function), which quantifies how extreme the observed count is under the null model of noise-driven peaks. The resulting p-values were then corrected for multiple comparisons across parcels using FDR.

This procedure, adapted from previous work [1], provides a principled way to threshold the Oscillatory Peak Count maps by evaluating whether the across-subject consistency of peak detection exceeds what would be expected from false positives alone (Supplementary Fig S3).

1.4 Effect of analyzing the power dynamics within canonical frequency range

LSD induces both a desynchronization of oscillatory activity and an acceleration of the alpha and beta rhythms. A key methodological contribution of this work is the mitigation of confounds that arise when power is analyzed strictly within canonical frequency bands, without accounting for drug-induced peak shifts. To address this, we quantify oscillatory amplitude using Peak Power, which extracts the power specifically at the individualized peak frequency. This approach isolates genuine oscillatory changes from those driven by shifts in peak location.

Panels (a) and (b) in Supp. fig. S4 show statistically thresholded contrast maps (using the same permutation-based pipeline as in the main text) for alpha and beta Peak Power, respectively. Significant contrast maps (same statistical pipeline as in the main text) for alpha (a) and beta (b) are presented with $1/f$ (first row) and without (second row).

1.5 Band-specific LZC reveals increased complexity in beta and low-gamma

We computed LZC for each frequency band starting with alpha, going up to high-gamma (Supp. Figure. S5).

1.6 Relations of the temporal and spectral features with Phenomenological dimensions at the global scale

How changes in the neurophysiological features relate to phenomenology is an important question that we addressed by regression analyses. To begin with, we assessed how the changes in the features *global level* i.e., region-averaged, relate to six phenomenological dimensions : 1) Simple Imagery; 2) Complex Imagery; 3) Intensity; 4) Emotional Arousal; 5) Positive mood; 6) Ego Dissolution. These analyses have been conducted for various statistical conditions: Main Effect Drug, Main Effect Music, Interaction Effects, Simple Effect Music, Simple Effect NoMusic. The corresponding heatmaps are presented in the supplementary material (Supplementary Figure S6). The raw phenomenological scores

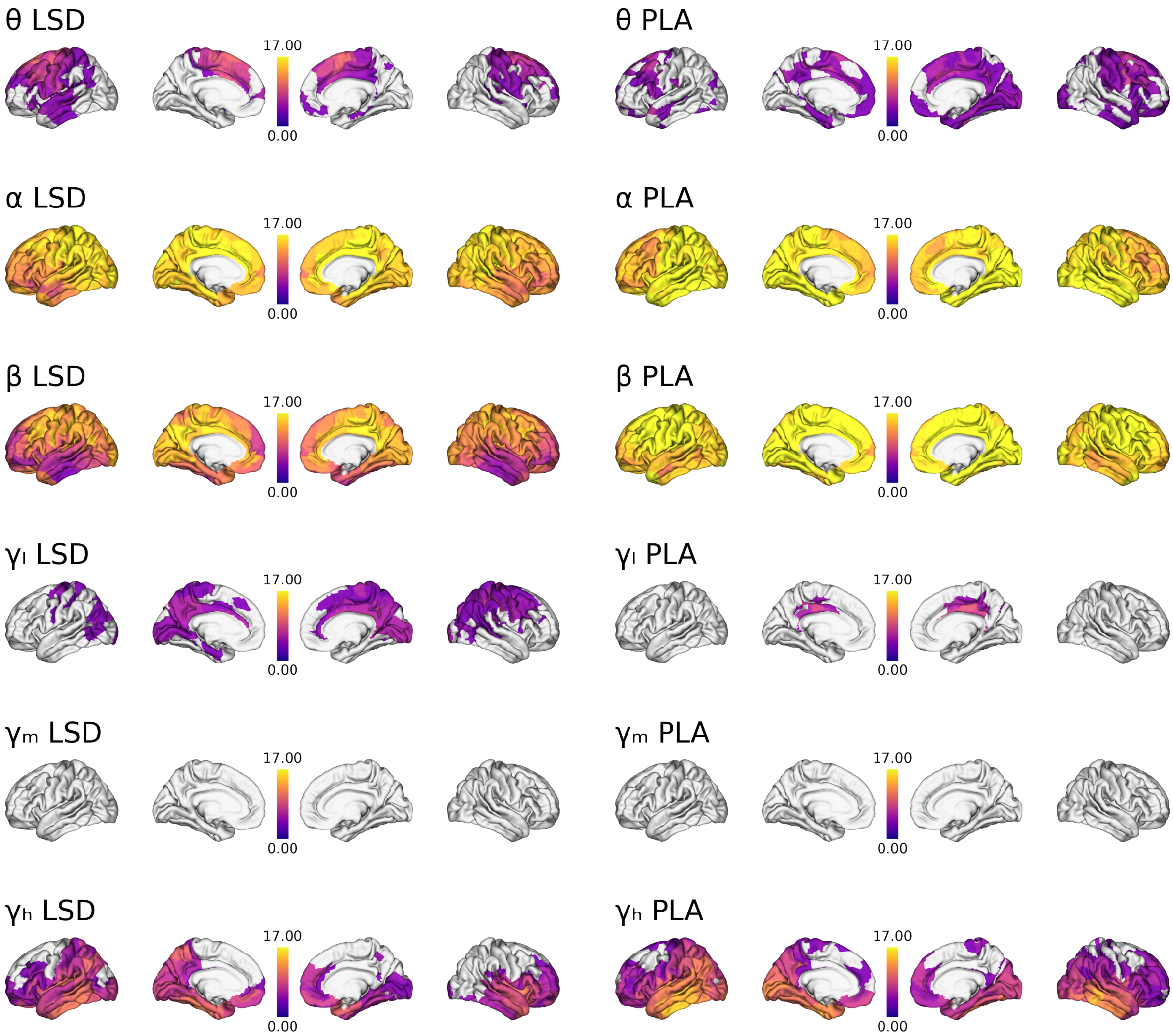

Figure S3: Oscillatory peak counts across frequency bands and drug conditions. For each cortical region, color intensity reflects the number of participants exhibiting a statistically significant oscillatory peak ($p < 0.05$, FDR-corrected) across θ, α, β, low-γ, mid-γ, and high-γ bands. Left and right halves of each row show peak-count maps for the LSD and placebo (PLA) conditions, respectively. Warmer colors indicate a greater number of participants with detectable peaks in that band.

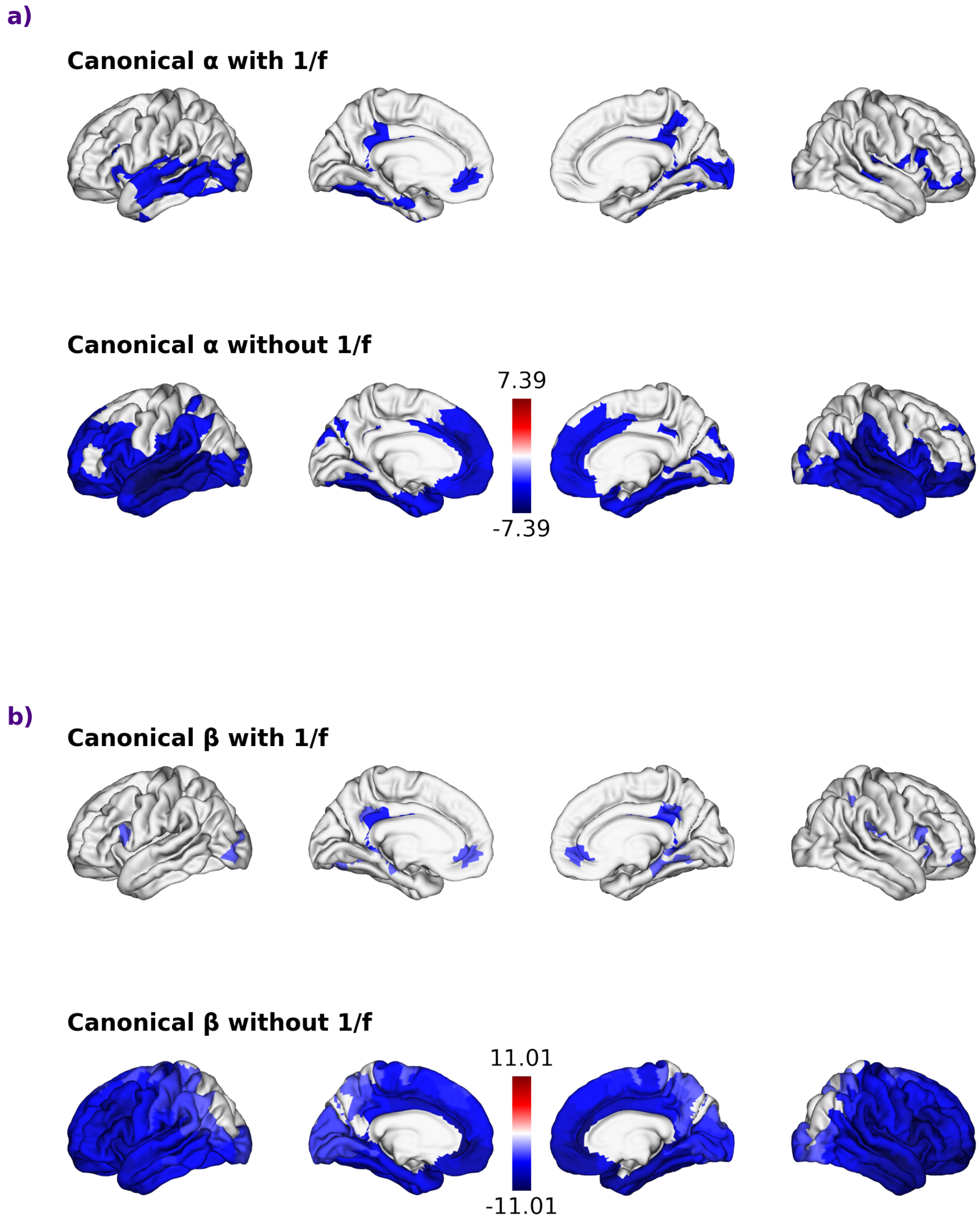

Figure S4: Power changes when analyzing with peak shifts. **a-b** Region-resolved changes in bandlimited power between LSD and PLA ($p<0.05$; permutation-corrected). For each band, the top row shows canonical band-power differences with the aperiodic ($1/f$) component included, and the bottom row shows differences after removing the $1/f$ component (oscillatory-only power).

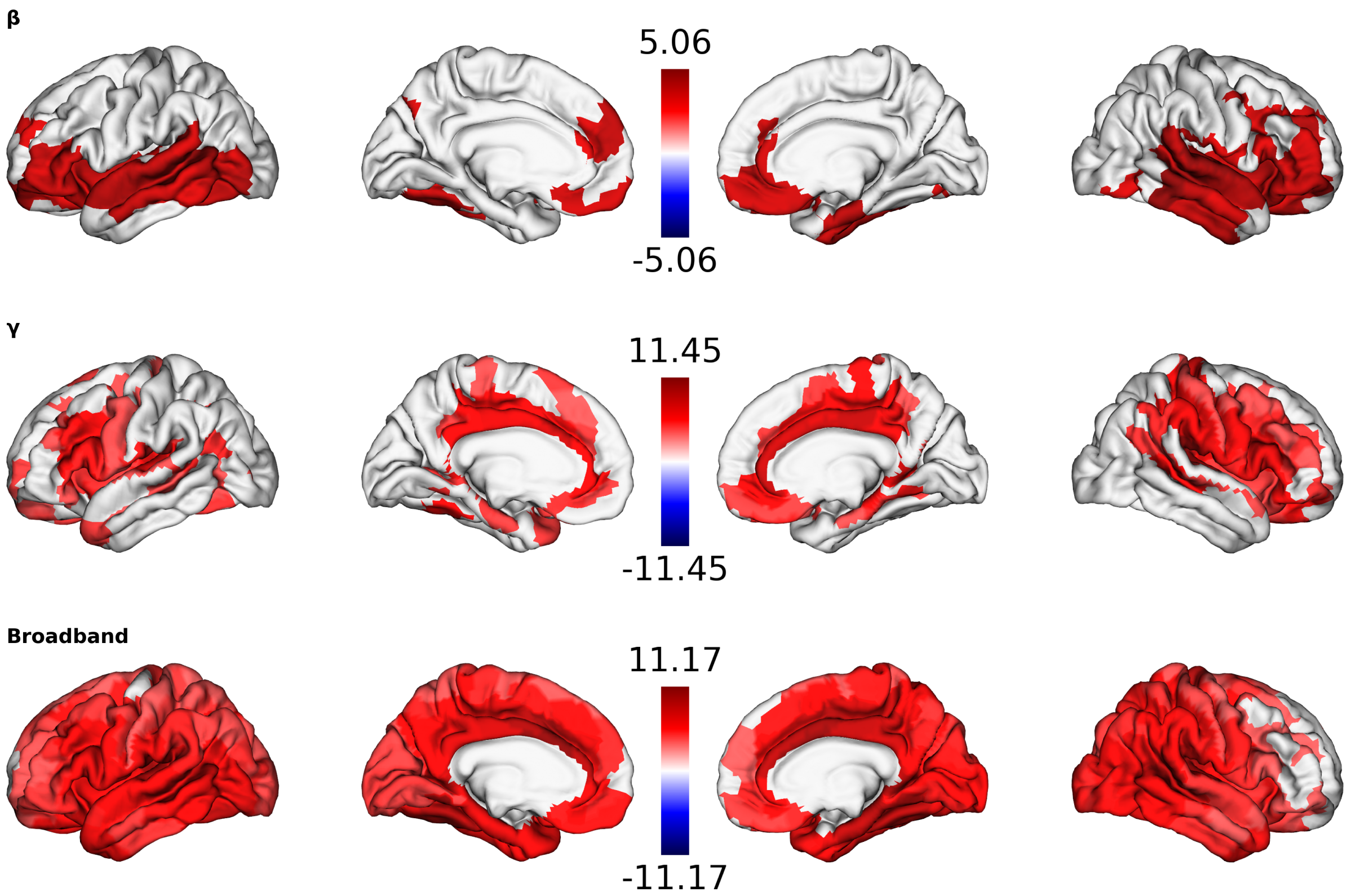

Figure S5: Group-level differences in adaptive Lempel-Ziv Complexity. For context, the significant (permutation-corrected, N=50,000, $p<0.05$) map for broadband signal (as in manuscript) is on the left. LZC computed on the filtered recordings showing significance in beta and low-gamma activity, while showing no significance in alpha, mid- and high-gamma.

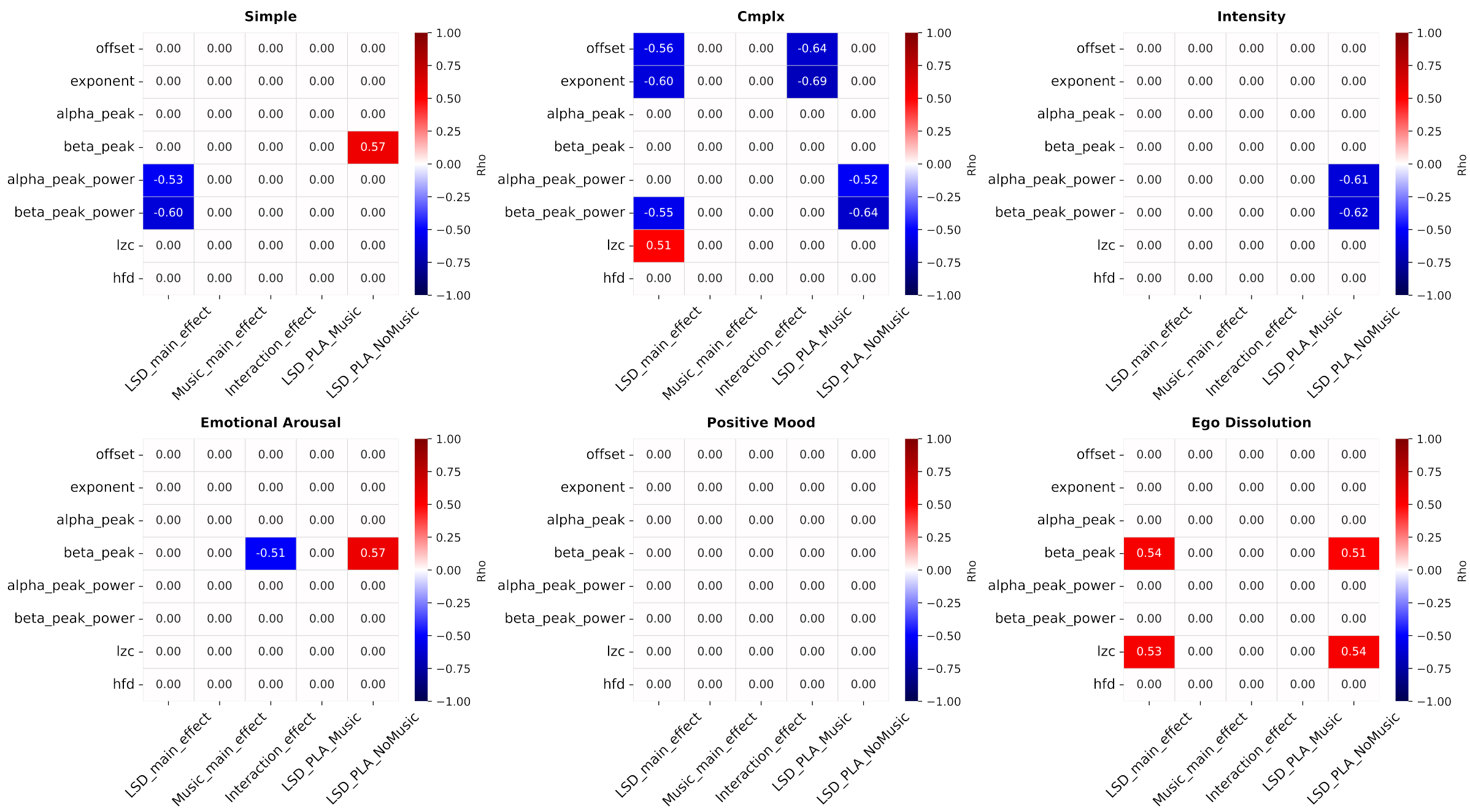

Figure S6: Global-level Regression analysis between electrophysiological features and Phenomenology.

are provided also in the supplementary material (Supplementary Figure S5). Spearman's ρ characterizes the level of correlation between the feature and the subjective ratings, ranging from -1 to 1. We can observe a tight link between feature phenomenology across many phenomenological dimensions. For example, flattening of the slope, and lowering of the offset were significantly associated with increased Complex Imagery. This trend was observed under the Main Effect Drug, and Simple Effect Music contexts. Similarly, alpha and beta Peak Power attenuation significantly correlated with drug's Intensity under Simple Effect NoMusic. In addition, increased LZC was associated with increased Complex Imagery under the Main Effect Drug. This gives insights about the trend at the global level.

1.7 Proportion of Regions with Significant Regressions by Phenomenology Dimension

We also investigated the region-level dynamics by performing regression analyses at a finer spatial scale (ROI-level). To this end, we ran Spearman's ρ tests across all the regions (N=360), and the significant ($p < 0.05$; FDR-corrected) correlation results are presented in the supplementary material (Supplementary Figure S7). Proportion of the significant correlation ($p < 0.05$, FDR-corrected) following the Spearman's ρ comparing the changes in neurophysiology at the individual region level and Phenomenology. Units here represent proportions (%) i.e. how many regions are significantly linked to phenomenology out of all regions. Main Effect Drug and Interaction effects are the conditions of interest. Shifts in beta peaks significantly correlate with increased imagery (both simple and complex) in less than 5% of the regions.

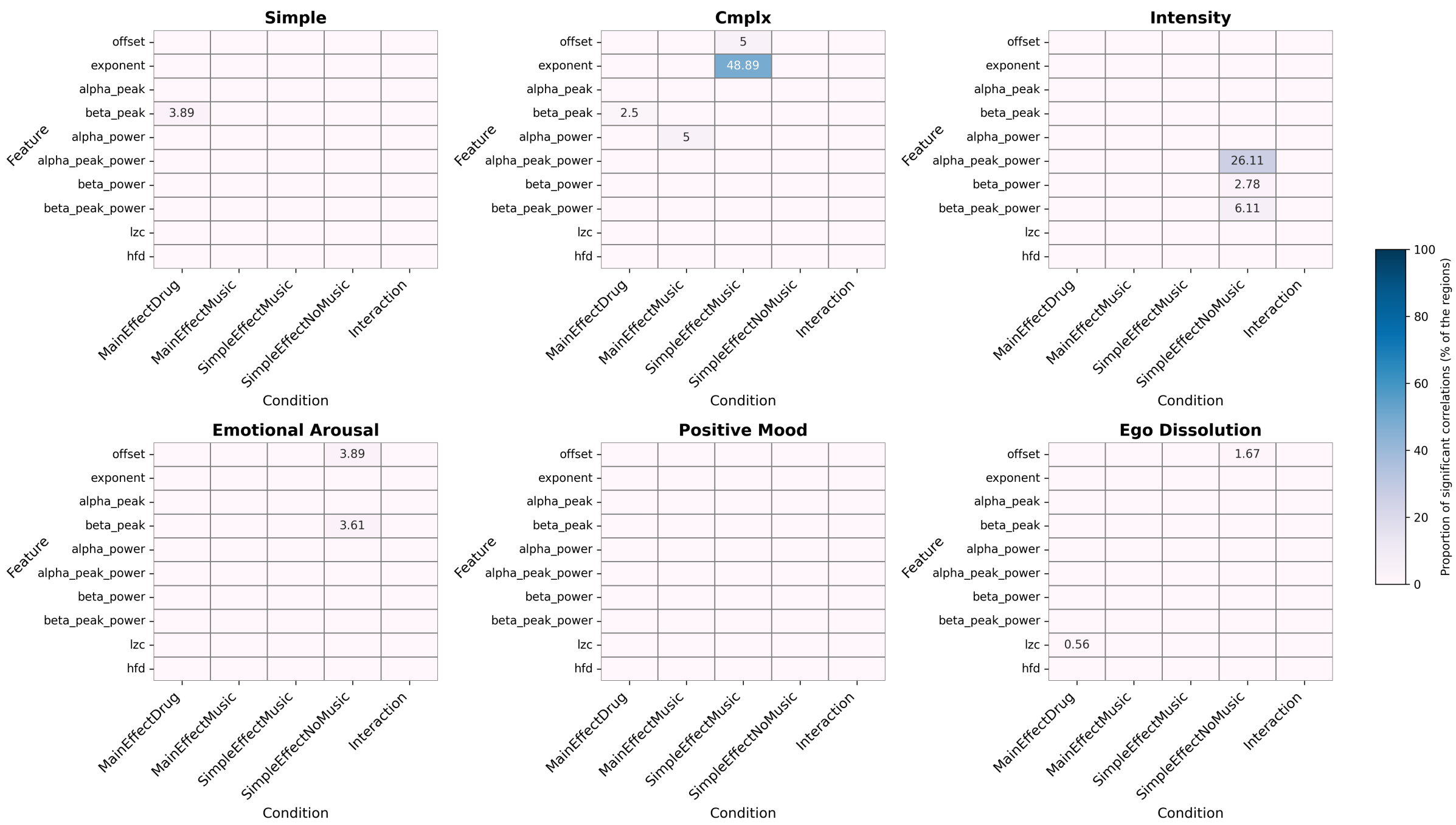

Figure S7: Proportion of Regions with Significant Regressions by Phenomenology Dimension.

1.8 AUC-ROC reveals strong sensitivity and specificity of the ML models

In addition to reporting the accuracy and feature importances of the ML models, we report here the AUC-ROC (Supp. Figure. S8).

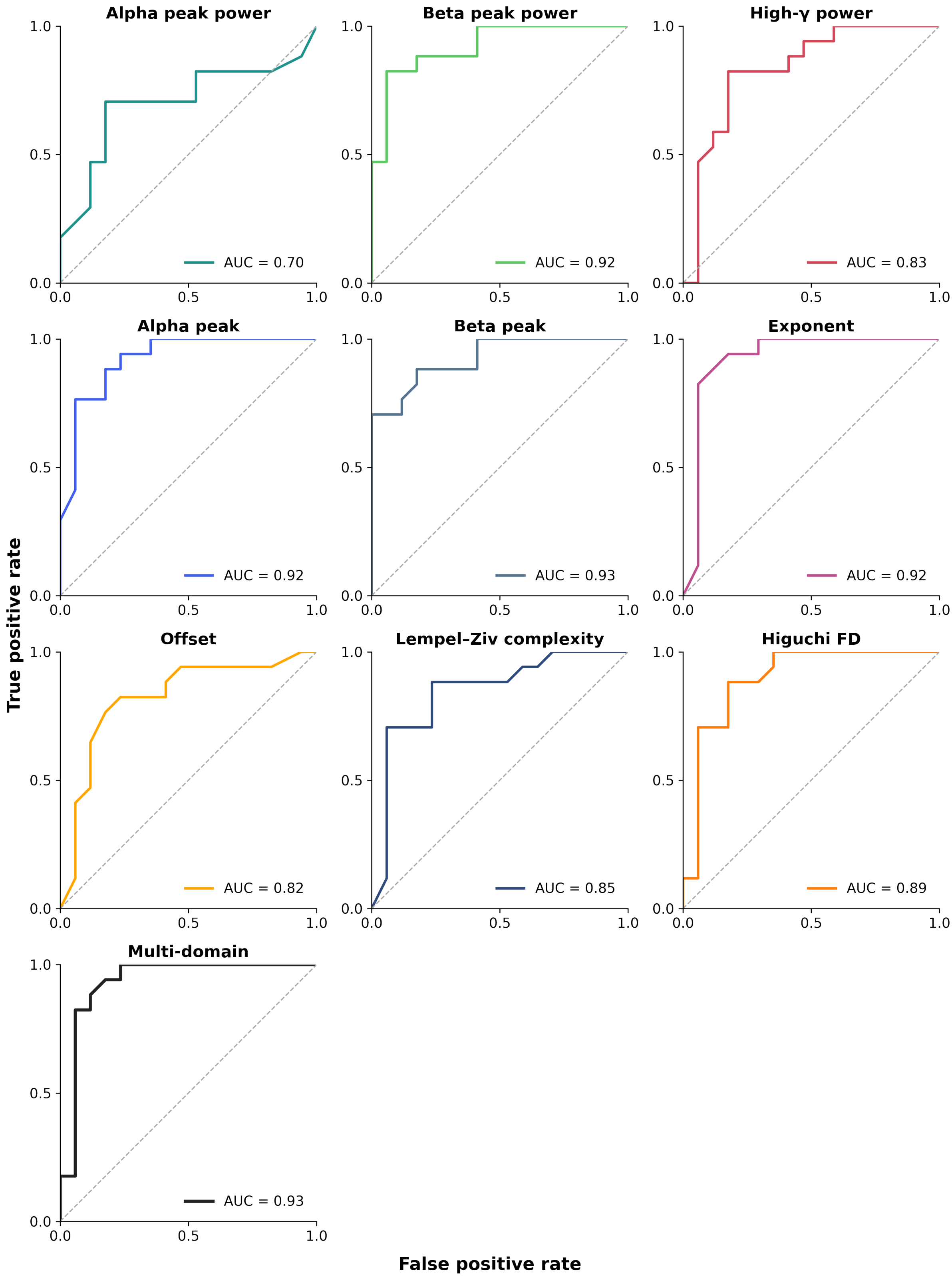

Figure S8: Receiver Operating Characteristic curve of the single-domain and multi-domain ML models. Y axis represents sensitivity and X the false-alarm (1 - Specificity)
