# Supplementary material for: LSD reconfigures cortical dynamics through faster brain rhythms and increased fractal dimension
Source: Commun Biol. 2026 Oct 7;9:1244. doi: 10.1038/s42003-026-10629-7 (PMC13646281; doi:10.1038/s42003-026-10629-7)
Supplement: Supplementary file 2 [file 42003_2026_10629_MOESM2_ESM.pdf]

nature portfolio

Corresponding author(s): Venkatesh Subramani & Karim Jerbi

Last updated by author(s): May 19, 2026

Reporting Summary

Nature Portfolio wishes to improve the reproducibility of the work that we publish. This form provides structure for consistency and transparency in reporting. For further information on Nature Portfolio policies, see our Editorial Policies and the Editorial Policy Checklist.

Statistics

For all statistical analyses, confirm that the following items are present in the figure legend, table legend, main text, or Methods section.

n/a	Confirmed	
☐	☒	The exact sample size (n) for each experimental group/condition, given as a discrete number and unit of measurement
☐	☒	A statement on whether measurements were taken from distinct samples or whether the same sample was measured repeatedly
☐	☒	The statistical test(s) used AND whether they are one- or two-sided<br>*Only common tests should be described solely by name; describe more complex techniques in the Methods section.*
☐	☒	A description of all covariates tested
☐	☒	A description of any assumptions or corrections, such as tests of normality and adjustment for multiple comparisons
☐	☒	A full description of the statistical parameters including central tendency (e.g. means) or other basic estimates (e.g. regression coefficient) AND variation (e.g. standard deviation) or associated estimates of uncertainty (e.g. confidence intervals)
☐	☒	For null hypothesis testing, the test statistic (e.g. F, t, r) with confidence intervals, effect sizes, degrees of freedom and P value noted<br>*Give P values as exact values whenever suitable.*
☒	☐	For Bayesian analysis, information on the choice of priors and Markov chain Monte Carlo settings
☒	☐	For hierarchical and complex designs, identification of the appropriate level for tests and full reporting of outcomes
☐	☒	Estimates of effect sizes (e.g. Cohen's d, Pearson's r), indicating how they were calculated

Our web collection on statistics for biologists contains articles on many of the points above.

Software and code

Policy information about availability of computer code

Data collection	The MEG data analysed in this study were collected by Carhart-Harris et al. (2016) using a CTF-275 whole-head MEG system sampled at 1200 Hz (0–300 Hz hardware bandpass). The present study is a secondary analysis; no new data were collected. Structural T1-weighted MRI scans accompanying the dataset were processed using FreeSurfer v7.4.1 (https://surfer.nmr.mgh.harvard.edu).
Data analysis	All analyses were performed in Python. MEG preprocessing, source localisation, and permutation-based statistics were implemented using MNE-Python v1.8 (https://mne.tools). Automated artefact rejection was performed using Autoreject (https://autoreject.github.io). ICA decomposition used the Picard algorithm as implemented in MNE. Spectral parameterisation was performed using the FOOOF/specparam toolbox v1.0 (https://fooof-tools.github.io). Higuchi fractal dimension was computed using the antropy package (https://raphaelvallat.com/antropy). Lempel–Ziv complexity was computed using a custom implementation. Machine-learning analyses used scikit-learn (https://scikit-learn.org). Functional decoding was performed using NiMARE v0.0.13 (https://nimare.readthedocs.io). All custom analysis code is publicly available at https://github.com/venkateshness/specparam_LSD.

For manuscripts utilizing custom algorithms or software that are central to the research but not yet described in published literature, software must be made available to editors and reviewers. We strongly encourage code deposition in a community repository (e.g. GitHub). See the Nature Portfolio guidelines for submitting code & software for further information.

Data

Policy information about availability of data

All manuscripts must include a data availability statement. This statement should provide the following information, where applicable:

- Accession codes, unique identifiers, or web links for publicly available datasets
- A description of any restrictions on data availability
- For clinical datasets or third party data, please ensure that the statement adheres to our policy

The MEG dataset analysed in this study was originally collected by Carhart-Harris et al. (2016, PNAS; https://doi.org/10.1073/pnas.1518377113) and is not publicly archived. Enquiries regarding data access should be directed to S.M. (s.muthukumaraswamy@auckland.ac.nz) and R.C.-H. (robin.carhart-harris@ucsf.edu). Analysis code reproducing all reported results is available at https://github.com/venkateshness/specparam_LSD.

Research involving human participants, their data, or biological material

Policy information about studies with human participants or human data. See also policy information about sex, gender (identity/presentation), and sexual orientation and race, ethnicity and racism.

Reporting on sex and gender	Not used as covariates in the present analyses (demographic details are in the original publication - Carhart-Harris et al. (2016)).
Reporting on race, ethnicity, or other socially relevant groupings	No race, ethnicity, or other socially relevant grouping variables were used as covariates in the present study. These variables are described in Carhart-Harris et al. (2016).
Population characteristics	Participants were healthy adult volunteers with prior experience of psychedelic drugs, as described in Carhart-Harris et al. (2016). Key inclusion criteria included absence of personal or first-degree family history of psychotic or serious psychiatric disorders, no current psychiatric diagnosis, and no use of contraindicated medications. Participants were screened medically prior to enrolment. Detailed covariate-level demographic data (age range, sex distribution) are reported in the original publication.
Recruitment	Participants were recruited by Carhart-Harris et al. (2016). Recruitment criteria are detailed in the original publication. The current study reanalysed the existing dataset.
Ethics oversight	The original study was approved by the National Research Ethics Service Committee London–West London, conducted under the revised Declaration of Helsinki (2000), ICH-GCP guidelines, and the NHS Research Governance Framework. Imperial College London sponsored the research, conducted under a Home Office Schedule 1 drug licence.

Note that full information on the approval of the study protocol must also be provided in the manuscript.

Field-specific reporting

Please select the one below that is the best fit for your research. If you are not sure, read the appropriate sections before making your selection.

☒ Life sciences ☐ Behavioural & social sciences ☐ Ecological, evolutionary & environmental sciences

For a reference copy of the document with all sections, see nature.com/documents/nr-reporting-summary-flat.pdf

Life sciences study design

All studies must disclose on these points even when the disclosure is negative.

Sample size	N = 17 participants. All 17 subjects went through LSD and Placebo sessions
Data exclusions	At the epoch level, bad epochs were identified and rejected via visual inspection and two passes of Autoreject. Mean epochs retained per subject per run: ~200 (SD=20). Epoch retention did not differ significantly between LSD and placebo conditions (paired t-test, t=−1.69, p<0.12). ICA-based rejection removed components reflecting cardiac, ocular, and muscular artefacts.
Replication	Replication of the spectral parameterization approach was performed within-dataset across two distinct frequency range fits (1–120 Hz and 1–45 Hz) to assess robustness.
Randomization	The original study used a within-subject, counterbalanced, single-blind design. LSD and placebo sessions were separated by 14 days to prevent drug carryover. The order of drug/placebo administration was counterbalanced across participants.
Blinding	Single-blind crossover design

Reporting for specific materials, systems and methods

We require information from authors about some types of materials, experimental systems and methods used in many studies. Here, indicate whether each material, system or method listed is relevant to your study. If you are not sure if a list item applies to your research, read the appropriate section before selecting a response.

Materials & experimental systems

n/a	Involved in the study
☒	☐ Antibodies
☒	☐ Eukaryotic cell lines
☒	☐ Palaeontology and archaeology
☒	☐ Animals and other organisms
☒	☐ Clinical data
☒	☐ Dual use research of concern
☒	☐ Plants

Methods

n/a	Involved in the study
☒	☐ ChIP-seq
☒	☐ Flow cytometry
☒	☐ MRI-based neuroimaging

Plants

Seed stocks	*Report on the source of all seed stocks or other plant material used. If applicable, state the seed stock centre and catalogue number. If plant specimens were collected from the field, describe the collection location, date and sampling procedures.*
Novel plant genotypes	*Describe the methods by which all novel plant genotypes were produced. This includes those generated by transgenic approaches, gene editing, chemical/radiation-based mutagenesis and hybridization. For transgenic lines, describe the transformation method, the number of independent lines analyzed and the generation upon which experiments were performed. For gene-edited lines, describe the editor used, the endogenous sequence targeted for editing, the targeting guide RNA sequence (if applicable) and how the editor was applied.*
Authentication	*Describe any authentication procedures for each seed stock used or novel genotype generated. Describe any experiments used to assess the effect of a mutation and, where applicable, how potential secondary effects (e.g. second site T-DNA insertions, mosiacism, off-target gene editing) were examined.*
