# Supplementary material for: LSD reconfigures cortical dynamics through faster brain rhythms and increased fractal dimension
Source: Commun Biol. 2026 Oct 7;9:1244. doi: 10.1038/s42003-026-10629-7 (PMC13646281; doi:10.1038/s42003-026-10629-7)
Supplement: Supplementary file 3 [file 42003_2026_10629_MOESM3_ESM.pdf]

nature portfolio

Peer Review File

LSD Reconfigures Cortical Dynamics Through Faster Brain Rhythms and Increased Fractal Dimension

Corresponding Author: Professor Karim Jerbi

This file contains all reviewer reports in order by version, followed by all author rebuttals in order by version.

Version 0:

Reviewer comments:

Reviewer #1

(Remarks to the Author)
The manuscript "LSD Reconfigures Cortical Dynamics Through Faster Brain Rhythms and Increased Fractal Dimension" by Venkatesh Subraman et al. provide a comprehensive electrophysiological characterization of how LSD reorganizes human cortical. Results show that LSD induces robust, spatially structured increases in alpha and beta peak frequencies. They also found genuine attenuation of oscillatory power, with these effects displaying partly dissociable cortical patterns. Beyond rhythmic activity. They further demonstrated LSD is associated with flattening of the aperiodic 1/f spectral 1 2 slope and increased neural signal fractality and complexity, preferentially affecting sensory, language, emotion, and imagery-related networks while sparing motor cortex. They present novel results for an interesting issue. The methodology and analysis are well suited for this study. The conclusions are correct and the discussion is comprehensive.
Nevertheless, this reviewer recommends enriching the discussion by comparing their results with LSD against other drugs like 5-MeO DMT that has similar mechanisms of action and other drugs that produce similar effects like ketamine, psilocybin, ETC.
This reviewer also suggests taking account of the next aspects.
• LSD increases on high gamma power only were evident in raw spectrum but disappeared in the oscillatory component; it could be that the aperiodic component of high gamma activity has a muscular origin. The authors discussed this in a comprehensive way, but this reviewer suggest seeking if LSD effects increase muscular activity. This can be done in a simple experiment by recording muscular activity under LSD effect and comparing it to the control state. If there are no differences, it is unlikely that the difference has muscular origin.
• When you analyze Lempel–Ziv complexity, your result shows the predominant effects off the higher power activity in this case alpha frequency. Since alpha activity tends to be homogeneous, a reduction in reduction in Lempel–Ziv complexity is expected in alpha power. To further study that aspect this reviewer suggests analyzing Lempel–Ziv complexity of filtered recordings showing only beta and higher frequencies to evidence if Lempel–Ziv complexity also increases for them. This allows to determinate the complexity of different frequencies.

Reviewer #2

(Remarks to the Author)
In this paper, the authors analyze MEG data and calculate spectral measures such as oscillatory powers and their frequencies as well as Lempel-Ziv complexity and fractal dimension to analyze the effects of LSD administration. They use permutation statistics along with machine learning to establish that LSD induces increases in alpha and beta peak frequencies and a decrease in their power. The findings are timely and technically sound. My brief comments are as follows:

1) The interaction effect between music and LSD seems to be very weak. The authors acknowledge that the result is nominally significant (only when p values are not corrected) and indicate this in the discussion. Nonetheless, given that one would need 16 times the (https://statmodeling.stat.columbia.edu/2018/03/15/need16/) sample size to get the same effect from an interaction compared to main effect, I would recommend indicating this weakness further in the discussion and limitations.

2) As far as I can tell from the manuscript, the machine learning models (random forest implementation) are not tested in a test data, where the model was not trained on. I can understand that low sample size (n=17) makes this hard. However, the

fact that accuracies are not too high (~0.7-0.8) is concerning. I can at least suggest doing a leave-one-out cross validation, along with ROC curves to see if the accuracy results hold up in test data.

3) What is the motivation behind using a random forest classifier instead of something simpler, such as logistic regression?

4) Authors say that "Consistent with this
interpretation, the absence of significant correlation between peak-power reductions and
peak-frequency shifts ($p > 0.05$) supports the view that these effects do not arise from
single mechanism." (page 23). To my knowledge, the absence of a significant correlation does not indicate a finding. It just tells something about repetition of the experiments. I would suggest authors to use the correlation coefficient here, which is a phsyiologically meaningful quantity.

Version 1:

Reviewer comments:

Reviewer #1

(Remarks to the Author)
The authors have adequately answered to my comments and appropriately addressed all concerns raised with the initial manuscript submission. I have no more suggestions to make.

Reviewer #2

(Remarks to the Author)
The authors have adequately addressed my comments. I have no further comments and can recommend the paper for publication.

Open Access This Peer Review File is licensed under a Creative Commons Attribution 4.0 International License, which permits use, sharing, adaptation, distribution and reproduction in any medium or format, as long as you give appropriate credit to the original author(s) and the source, provide a link to the Creative Commons license, and indicate if changes were made.
In cases where reviewers are anonymous, credit should be given to 'Anonymous Referee' and the source.
The images or other third party material in this Peer Review File are included in the article’s Creative Commons license, unless indicated otherwise in a credit line to the material. If material is not included in the article’s Creative Commons license and your intended use is not permitted by statutory regulation or exceeds the permitted use, you will need to obtain permission directly from the copyright holder.
To view a copy of this license, visit https://creativecommons.org/licenses/by/4.0/
